# Comparison of Liver Biopsy Findings with the Digestive Disease Week Japan 2004 Scale for Diagnosis of Drug-Induced Liver Injury

**DOI:** 10.1155/2015/913793

**Published:** 2015-11-01

**Authors:** Akemi Tsutsui, Yasuni Nakanuma, Kouichi Takaguchi, Satoko Nakamura, Hiroshi Shibata, Nobuyuki Baba, Tomonori Senoh, Takuya Nagano, Hiroko Ikeda

**Affiliations:** ^1^Department of Hepatology, Kagawa Prefectural Central Hospital, Kagawa 760-8557, Japan; ^2^Department of Pathology, Shizuoka Cancer Center, Shizuoka 411-0934, Japan; ^3^Department of Human Pathology, Kanazawa University Graduate School of Medicine, Kanazawa 920-0934, Japan; ^4^Section of Diagnostic Pathology, Kagawa Prefectural Central Hospital, Kagawa 760-8557, Japan; ^5^Department of Gastroenterology, Tokushima Prefectural Central Hospital, Tokushima 770-0042, Japan; ^6^Section of Diagnostic Pathology, Kanazawa University Hospital, Kanazawa 920-0934, Japan

## Abstract

The liver biopsy remains a valuable tool in the diagnosis of drug-induced liver injury (DILI). The Digestive Disease Week Japan 2004 (DDW-J) scale proposed as an objective tool for the diagnosis of DILI has been widely used in Japan. So far, the histological features have not been compared with DDW-J scale in detail. Herein, we examined the correlation between liver biopsy findings and clinical features, particularly DDW-J scales. A total of 80 patients with liver injuries of unknown cause were enrolled. Based on the histological findings, these cases were categorized into 3 groups: A (DILI was strongly suspected), B (DILI was suspected), and C (DILI should be considered in the differential diagnosis). Histological groups and DDW-J scale were moderately correlated (*κ* = 0.60). The mean total DDW-J scale scores were as follows: 4.89 for A, 3.26 for B, and 0.75 for C (*p* < 0.05). While hepatocellular type was coincided in a majority of cases by histological and DDW-J scale evaluation, cholestatic type was not well coincided. In conclusion, biopsy findings and DDW-J scale were well correlated, and the hepatocellular type of liver injuries was well coincided by both evaluations, though there were several discrepant cases, particularly in cholestatic type.

## 1. Introduction

Drug-induced liver injury (DILI) is an important cause of liver injuries with significant morbidity and mortality [1, 2]. Accurate and early diagnosis is important but the diagnosis of DILI is complicated and nonstandardized because of the difficulty in identification drug(s) causing liver injuries and lack of reliable markers to facilitate and establish a diagnosis of DILI [3, 4]. The most important factors to be considered in the diagnosis of drug-induced liver injury (DILI) are the time relationship between the drug administration and appearance/disappearance of liver injury, and the exclusion of other potential causes. Recently, clinical scales or scores have developed to facilitate a diagnosis of DILI. The Council for International Organizations of Medical Sciences/the Roussel Uclaf Causality Assessment Method (CIOMS/RUCAM) scale was proposed [5] and has been generally used as a standardized diagnostic tool. In Japan, the Digestive Disease Week Japan 2004 (DDW-J) scale, which is highly sensitive and specific, was developed by modifying the CIOMS/RUCAM scale [6–8]. This DDW-J scale was proposed as an objective tool for the diagnosis of DILI and has been widely used in Japan [9].

Despite the associated limitations and aggressive diagnostic tool, the liver biopsy remains still a valuable tool in the evaluation of patients suspected to have DILI and can provide valuable information for the diagnosis and management of DILI. That is, when the cause of the liver injury is not apparent, liver biopsy can indicate the possible presence of DILI and can enable an assessment of the histological patterns by referring to known histological patterns related to potential drugs. Second, liver biopsy can contribute to the management of DILI by assessing the severity and histological features of liver injury.

However, the histologic findings of liver biopsy alone are often insufficient for confirming a diagnosis of DILI, as the diverse histological patterns can mimic any primary liver disease [5, 10–13]. Therefore, clinical investigations are also essential to make a definite diagnosis of DILI. While the comparison of the histological features of liver biopsies and DDW-J scale in the patients with a suspected diagnosis of DILI seems important, detailed correlational studies have not been performed, so far.

In this study, we investigated the relationship between pathological findings of liver biopsies and clinical features, particularly DDW-J scale for the diagnosis of DILI, by using 80 patients with liver biopsy findings showing a suspicion of DILI.

## 2. Materials and Methods

### 2.1. Selection of Patients and Tissue Preparation

Subjects were selected from 2115 patients who underwent liver biopsy, when the liver biopsy examination indicated a potential presence of DILI, in two hospitals (Kanazawa University Hospital and Kagawa Prefectural Central Hospital) from April 2007 to December 2014. A total of 80 patients (35 men and 45 women), with a mean age of 55.0 years (range: 15 to 83 years), were enrolled in this study. Clinical and laboratory data were obtained from medical records in these hospitals.

Liver biopsy specimens obtained from these patients were fixed in 10% formalin and embedded in paraffin and were processed routinely for histological diagnosis.

### 2.2. The Digestive Disease Week Japan 2004 (DDW-J) Scale

DDW-J scale was developed by modifying the CIOMS/RUCAM scale [6–8]. In particular, the factor of comedication was excluded, and the factors of drug lymphocyte stimulation test and eosinophilia were included according to Japan's clinical environment. Details regarding these two scales are summarized in Table 1. Each case was assessed according to DDW-J scale, Japanese clinical diagnostic criteria for DILI [9]. First, the cases were scored for 8 items indicated: time to onset, course, risk factors, other causes, previous information on hepatotoxicity, eosinophilia, drug-lymphocyte stimulation test, and response to readministration. Based on the total scores, individual cases were classified to 3 grades with respect to a diagnosis of DILI; 5 or more, probable; 3 to 4, possible; 2 or less, unlikely (Table 1(a)). Then, the cases were classified to the hepatocellular, cholestatic, and mixed hepatocellular and cholestatic type according to the serum levels of alanine aminotransferase (ALT) and alkaline phosphatase (ALP). Hepatocellular type was defined as ALT > 2 × ULN (upper limit of the normal range) and ALP≦ULN, or *R*≧5, where the *R* value was calculated as (ALT/ULN)/(ALP/ULN). Cholestatic type was defined as ALT≦ULN and ALP > 2 × ULN, or *R*≦2. Mixed type was defined as ALT > 2 × ULN, ALP > ULN, and *R* > 2 and <5.

Causality assessment of drug-induced liver injury (hepatocellular type) is as follows:

(1) time to onset (Table 1(b));
(2) course of the reaction, after cessation of the administration of the drug: score +3: the course is very suggestive if there is decrease of ALT≧50% of the excess over the upper limit of normal within 8 days. Score +2: the course is suggestive if there is decrease of ALT≧50% of the excess over the upper limit of normal within 30 days. Score 0: the course is inconclusive if there is decrease of ALT < 50% of the excess over the upper limit of normal within 30 days or no information regarding liver tests. Score −2: the course is not suggestive if there is decrease of ALT < 50% of the excess over the upper limit of normal after 30 days or increase of ALT again;
(3) in case of readministration of the drug, Score +3: the response is positive if there is at least a doubling of ALT irrespective of date, duration without another drug. Score +1: the response is compatible if there is at least a doubling of ALT irrespective of date, duration with another uninterrupted drug. Score 0: the response is uninterpretable under other conditions. Score −2: the response is negative if the increase is less than normal range, provided that the drug has been given in the same dose, for the same duration and with the same combined drugs as for the first administration.

Causality assessment of drug-induced liver injury (cholestatic type) is as follows:

(1) time to onset (Table 1(c));
(2) course of the reaction, after cessation of the administration of the drug: Score +2: the course is suggestive if there is decrease of ALP≧50% of the excess over the upper limit of normal within 180 days. Score +1: the course is intermediate if there is decrease of ALP < 50% of the excess over the upper limit of normal within 180 days. Score 0: the course is inconclusive if the levels are stable, and there is increase of ALP or no information regarding liver tests. If the drug is continued, the course is always inconclusive as regards causality assessment. Score −2: the course is not suggestive if there is decrease of ALP < 50% of the excess over the upper limit of normal after 30 days or increase of ALP again;
(3) in case of readministration of the drug, Score +3: the response is positive if there is at least a doubling of ALP irrespective of date, duration without other drug. Score +1: the response is compatible if there is at least a doubling of ALP irrespective of date, duration with other uninterrupted drug. Score 0: the response is uninterpretable under other conditions. Score −2: the response is negative if the increase is less than normal range, provided that the drug has been given in the same dose, for the same duration and with the same combined drugs as for the first administration.

### 2.3. Histological Examination

Histological examination was performed by three pathologists (Yasuni Nakanuma, Satoko Nakamura, and Hiroko Ikeda) with 20 years of experience of diagnostic liver pathology. DILI was suspected when at least one of the pathological features suggesting DILI, as shown in Box 1, was observed. Based on the histological findings, other potential causes of liver injuries were ruled out as far as possible. When a patient had a history of drug(s) administration with possibility to cause liver injury, the histological pattern(s) were examined by referring to the histopathologic patterns reported for individual drug(s) in the literatures. In this study, we retrospectively analyzed the pathological findings examined as described above.

**Figure 1:**
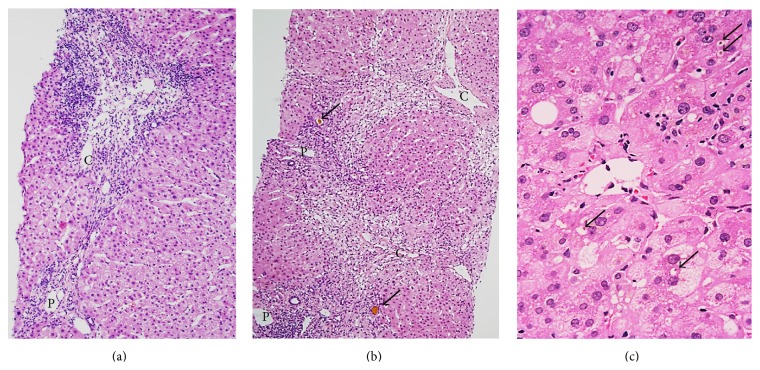
Typical histological images of DILI. (a) Hepatocellular type. Histological examination showed centrilobular hepatic necrosis with punched out lesions. The inflammatory changes were relatively mild in the portal areas, compared with those in the liver parenchyma. (b) mixed type. There is dropout of liver cells in the perivenular zone and bridges of necrosis (“central to portal”). Note periportal mild to moderate inflammatory response. There is dropout of liver cells in the perivenular zone. Additionally cholestasis is observed. (c) Cholestatic type. Ballooning degeneration of liver cells and marked cholestasis (needle biopsy, H&E). C: central vein, P: portal vein, and black arrows: bile thrombus.

Based on the comprehensive evaluation of histologic findings of liver, individual cases were categorized into three groups: group A (DILI is strongly suspected, when at least one of the pathological features suggesting DILI, as shown in Box 1, was observed and all pathologists suspected DILI), group B (DILI is suspected, when at least one of the pathological features suggesting DILI was observed), and group C (DILI should be considered in the differential diagnosis, when pathological features suggesting DILI were not observed) (Table 2). Patients who were finally diagnosed clinically with DILI (see below) were further histologically classified into three types: the hepatocellular type, cholestatic type, and mixed hepatocellular and cholestatic type [5]. The hepatocellular type was characterized by predominant necrotic and/or inflammatory changes of hepatic parenchyma including periportal regions, the cholestatic type by predominant canalicular cholestasis with or without other features of cholestasis, and the mixed type by considerable hepatocellular and cholestatic changes (Figure 1).

### 2.4. Final Clinical Diagnosis and Comparative Analyses between Histologic Groups and DDW-J Scale

The final clinical diagnosis of DILI was comprehensively made based on clinical course including laboratory and clinical findings with a help of consideration of histologic findings and also DDW-J scale for a diagnosis of DILI. Then, the final clinical diagnosis of DILI and the histological grouping were compared. The DDW-J scale scores and the histological groups were also compared. Furthermore, the histological patterns (hepatocellular type, cholestatic type, and mixed type) and clinical types according to the DDW-J scale (hepatocellular type, cholestatic type, and mixed type) were compared for the patients who were finally diagnosed as DILI.

### 2.5. Statistical Analysis

Statistical analysis was carried out using Student's *t*-test and weighted *κ* test. Differences were considered significant when the *p* value was less than 0.05.

## 3. Results

### 3.1. Histological Grouping and Final Clinical Diagnosis of DILI

**Figure 2:**
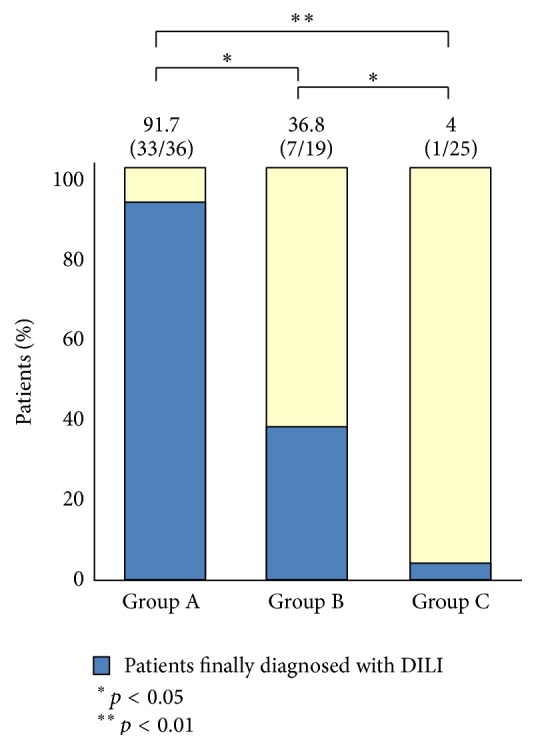
The histological examination results and the final diagnosis of DILI.

**Figure 3:**
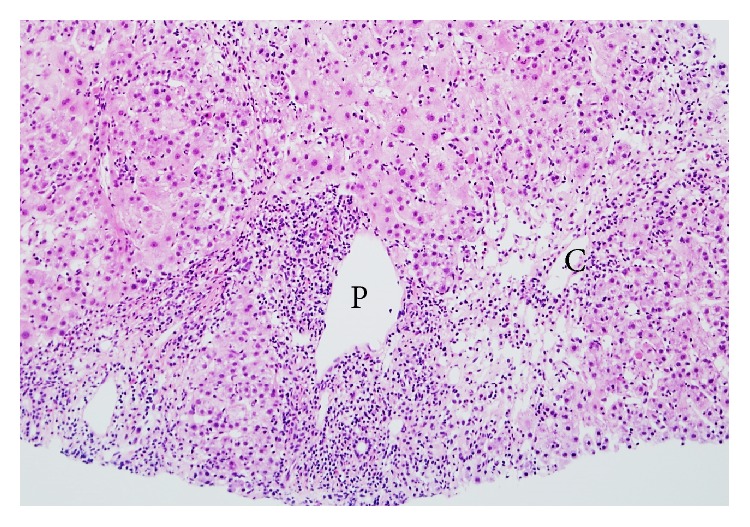
Autoimmune hepatitis. Histological examination showed centrilobular hepatic necrosis with punched out lesions, acidophilic body formation, enlargement of Kupffer cells, and sinusoidal lymphocyte infiltration. Moderate lymphocyte infiltration was observed in the portal areas. Eosinophilic infiltration was also observed. The histological findings were consistent with a diagnosis of acute severe hepatitis with confluent necrosis (needle biopsy, H&E). C: central vein; P: portal vein.

**Figure 4:**
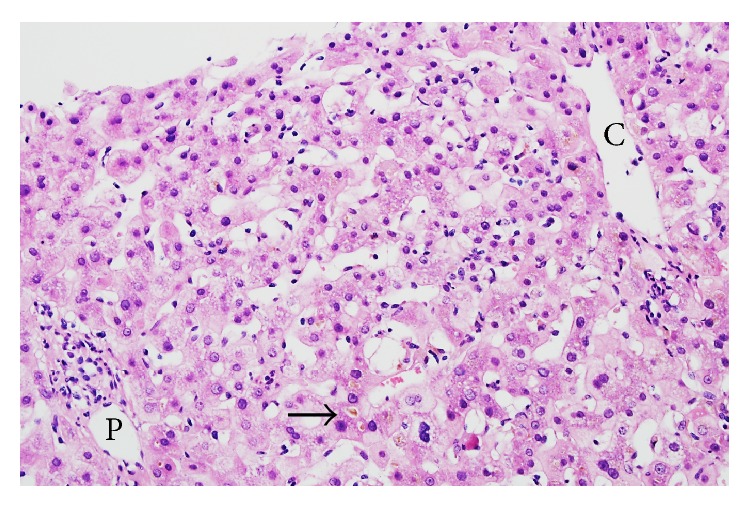
Hepatitis E. Mild lymphocyte infiltration was observed in the portal areas, but interface hepatitis was not present. Cholestasis and bile thrombus (black arrow) were clearly observed in the hepatocytes and predominantly in the periphery of the central vein (needle biopsy, H&E). C: central vein; P: portal vein.

By histological examinations, 36 patients were assigned to group A, 19 patients to group B, and 25 patients to group C. By comprehensive clinical and laboratory analyses of these 80 patients, a total of 41 patients were finally diagnosed with DILI. It was found that 33 of 36 (91.7%) patients in group A, 7 of 19 patients (36.8%) in group B, and 1 of 25 patients (4%) in group C were diagnosed finally with DILI (Figure 2). The incidence of final diagnosis of DILI was higher in group A compared to groups B (*p* < 0.05) and C (*p* < 0.01), and the incidence was also higher in group B than in group C (*p* < 0.05), suggesting a good correlation between the histological groups and the final clinical diagnosis of DILI. In 3 of 36 patients of group A, a diagnosis of DILI was eventually excluded: two patients had autoimmune hepatitis (AIH) (Figure 3) and one had graft versus host disease (GVHD). Among the 19 patients in group B, one patient was finally diagnosed with hepatitis E (Figure 4).

### 3.2. Correlation between Histologic Grouping and DDW-J Scale

**Figure 5:**
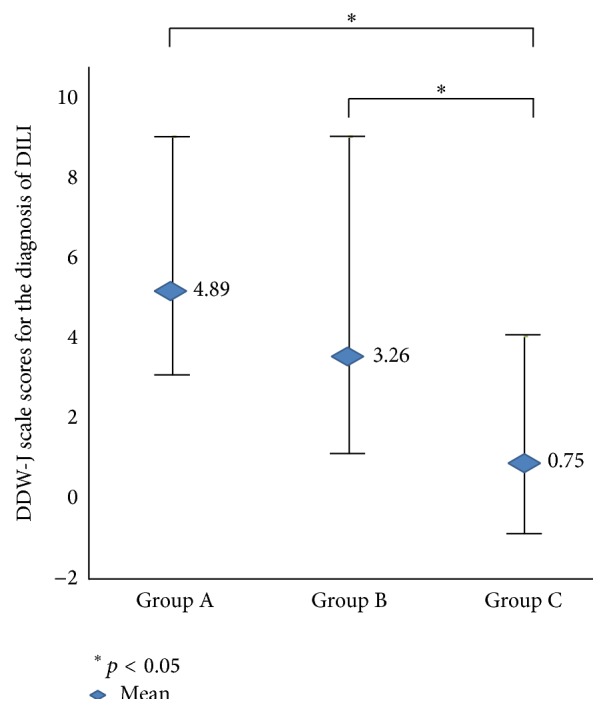
The histological examination results and DDW-J scale scores for the diagnosis of DILI.

Table 3 shows distribution of 80 cases with respect to histologic groups (A, B, and C) and DDW-J scale (probable, possible, and unlikely). Among the 41 patients with a final clinical diagnosis of DILI, 28 of 28 patients (100%) were classified as probable by DDW-J scale, suggesting a good correlation between final clinical diagnosis and DDW-J scale assessment. A majority of group A cases were diagnosed finally as probable DILI, while only 4 of 25 cases of group C were diagnosed as possible DILI and a majority of group C cases were diagnosed as unlikely DILI category. Group B cases were rather evenly distributed to probable, possible, and unlikely DILI. These two distributions were moderately correlated (*κ* = 0.60). The mean total DDW-J scale scores for the diagnosis of DILI in these three groups were as follows: 4.89 ± 1.96 for group A, 3.26 ± 1.95 for group B, and 0.75 ± 1.79 for group C (Figure 5): the scores were high in group A and lower in group C, and group B was between them, and there was statistical difference between groups A and C (*p* < 0.05) and also between groups B and C (*p* < 0.05), suggesting a good correlation between histological grouping and the scores of the DDW-J scale. There were no statistical differences between group A and group B.

### 3.3. Three Types of Liver Injuries of DILI by Histological Evaluation and by DDW-J Scales

Among the 41 patients with a final clinical diagnosis of DILI, 30 of 34 patients (73.2%) were classified as the hepatocellular type by histological evaluation and also DDW-J scale, suggesting a good correlation as for the hepatocellular type between histological and DDW-J scale assessment (Table 4).

Among these 41 patients, three patients were classified histologically as the cholestatic type. Of these three patients, one was classified as the cholestatic type and two were clinically classified as the hepatocellular type by DDW-J scale assessment. The latter two patients had higher ALT levels of ≥500, along with a high ALT/AST ratio. Although these two patients had elevated levels of ALP and T-bil, they were clinically classified as the hepatocellular type according to the diagnostic criteria (Table 5).

Among these 41 patients, four patients were clinically classified as the cholestatic type by DDW-J scale. Of the four patients, one was histologically classified as the cholestatic type and three were histologically classified as the hepatocellular type. Among the latter three patients, two had rheumatoid arthritis as an underlying disease; thus, an increase in the ALP level might have been bone-derived rather than hepatogenic, suggesting that ALP did not reflect the degree of cholestasis among these patients (Table 6).

## 4. Discussion

The data obtained in this study were summarized as follows. (i) Liver biopsy cases were classified into three groups according to a likelihood of DILI. (ii) Good correlation between histological groups and three grades of DILI based on the DDW-J scale was obtained. (iii) Among three patterns of liver injuries, the hepatocellular type was well coincided in both assessments, but cholestatic type was not well coincided. Taken together, liver biopsy findings and DDW-J scale were well correlated for diagnosis of DILI, and the hepatocellular type was well coincided, though there were several discrepant cases, particularly in cholestatic type. There might be some limits to classification of cholestatic type in DDW-J scale, because drug-induced cholestatic injury consisted mainly of bile accumulation in the cytoplasm of liver cells (hepatocellular cholestasis) and in canaliculi (canalicular cholestasis). We considered that liver biopsy was useful for diagnosis of DILI in cholestatic type.

In this study, we first categorized liver biopsy findings into three groups: group A (DILI is strongly suspected), group B (DILI is suspected), and group C (DILI should be considered in the differential diagnosis), and it was found that a good correlation was obtained between three groups of liver biopsy diagnosis and the final clinical diagnosis of DILI, suggesting that this grouping of liver biopsies seems useful in clinical practice. This grouping was comprehensively done by combination of histologic findings suggesting DILI (Box 1). However, the combination of pathological findings was different in individual cases and could not be formalized or subjected at the moment. Furthermore, the histological examinations are based on experiences of individual pathologists. More formalized categorization of liver biopsy evaluation for a diagnosis of DILI is necessary.

Recently, the Drug-Induced Liver Injury Network (DILIN) is an ongoing, multicenter observational study of consecutive cases of DILI enrolled at eight geographically distributed academic medical centers in the United States [14]. The central aims of the DILIN Network are to more fully characterize the clinical syndromes of liver injury caused by medications, herbals, and dietary supplements (HDS), to standardize terminology and grading systems, and to provide resources for mechanistic studies of DILI. Kleiner et al. have classified the pathological pattern of liver injury and systematically evaluated histological changes in liver biopsies obtained from 249 patients with suspected DILI enrolled in the prospective, observational study conducted by DILIN [15]. They described that adoption of a standardized and systematic approach to describe the histology of DILI will also allow for comparison of findings across studies and will help in standardizing management and providing insights into pathogenesis as well as approaches to therapy.

The Digestive Disease Week Japan 2004 (DDW-J) scale, which is highly sensitive and specific, was developed by modifying the CIOMS/RUCAM scale [6–8], was proposed as an objective tool for the diagnosis of DILI, and has been widely used in Japan [9]. Based on the total scores, individual cases were classified to 3 grades with respect to a diagnosis of DILI: probable, possible, and unlikely (Table 1(a)). It was found in this study that three groups of liver biopsy findings and three grades of DDW-J scales were well correlated. That is, the distribution of cases with respect to three grades of DDW-J scales and three groups of liver biopsies were moderately correlated, and the scores of DDW-J scales were higher in group A and lower in group C, and scores of group B were between them. However, there were also discrepant cases. In the manual of DDW-J scale, it is described that differential diagnoses of idiopathic autoimmune hepatitis (AIH) and DILI are difficult. In our study, among the 4 patients who were strongly suspected initially to have DILI based on the histological examination results (group A) but were not eventually diagnosed with DILI, two patients had AIH. These conditions might have been mediated by immunological reactions and thus show considerable resemblance in clinical and histopathologic features [16]. Suzuki et al. performed a standardized histologic evaluation to explore potential hallmarks to differentiate AIH versus DILI. The study showed that no single feature was indicative of AIH or DILI, but rather the combination of distinct findings, such as the types of inflammatory cells in different areas, severity of injury/inflammation, and presence of cholestasis, was very helpful in differentiating DILI versus AIH [17]. We considered that liver biopsy was useful in cases where the differential diagnoses were difficult.

HEV infection contributes to a small but important proportion of cases of acute liver injury that are suspected to be drug induced. In our study, among the 19 patients who were possibly suspected to have DILI based on the histological examination results (group B), one patient was finally diagnosed with hepatitis E. Also in Europe and the United States, there has been an increase in the prevalence of hepatitis E in individuals who had no history of overseas travel. Davern et al. examined the prevalence of hepatitis E virus in 318 patients who were previously diagnosed with DILI and reported that nine out of the 318 individuals were positive for the IgM hepatitis E virus antibody [18]. They said that hepatitis E should be considered in the differential diagnosis of patients with acute hepatitis of unknown cause. Although the patient in the present study showed changes suggestive of acute viral hepatitis, we suspected DILI because the changes in the portal areas were relatively mild compared with those in the liver parenchyma.

DILI is generally classified histologically into three types: hepatocellular, cholestatic, and mixed. DDW-J scale also proposed such classification into the hepatocellular, cholestatic, and mixed hepatocellular and cholestatic type according to the serum levels of alanine aminotransferase (ALT) and alkaline phosphatase (ALP). It was found in this study that a majority of hepatocellular type cases were coincided in both histological and laboratory assessment by DDW-J scale. However, the cholestatic cases were not coincided. As we discussed in Section 3, this discrepancy may be some limitations in DDW-J scale and more comprehensive clinical analysis of DILI cases is necessary in classification of liver injuries of DILI.

In conclusion, our study suggested a good correspondence between the histological groups with likelihood to a diagnosis of DILI and three grades of DDW-J scales. Hepatocellular type was well coincided by both liver biopsy and DDW-J scale, though cholestatic type was not well classified by DDW-scale. More objective and formalized grouping of liver biopsy findings and the exact classification of cholestatic type in DDW-J scale seem mandatory in a diagnosis of DILI.

## Figures and Tables

**Box 1:**
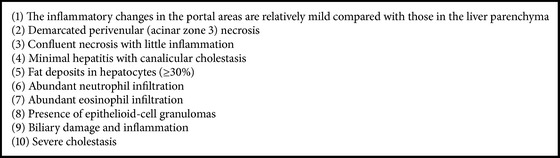
**Box 1: **Pathological features suggestive of DILI.

**(a):** 

Scales	DDW-J	CIOMS/RUCAM
Score		
Time to onset	0 to +2	0 to +2
Course of the reaction	−2 to +3	−2 to +3
Risk factor		
Age		0 to +1
Alcohol, pregnancy	0 to +1	0 to +1
Comedication		−3 to 0
Other causes^*∗*^	−3 to +2	−3 to +2
Previous information on hepatotoxicity	0 to +1	0 to +2
Eosinophilia	0 to +1	
Drug-lymphocyte stimulation test	0 to +2	
Response to readministration	−2 to +3	−2 to +3

Thresholds		
Highly probable		>8
Probable	≧5	6 to 8
Possible	3 to 4	3 to 5
Unlikely	≦2	1 to 2
Excluded		≦0

^*∗*^Score +2: all causes in groups I and II are ruled out. Score +1: all causes in group I are ruled out. Score 0: 5 or 4 causes in group I are ruled out. Score −1: less than 4 causes in group I are ruled out. Score −2: less than 3 causes in group I are ruled out. Score −3: nondrug cause highly probable.

Group I: HAV, HBV, HCV, biliary obstruction, alcoholism, and acute recent hypotension history. Group II: cytomegalovirus and Epstein-Barr virus.

**(b):** 

	Suggestive from onset of drug administration (score +2)	Compatible (score +1)		Incompatible (score 0)	
		From onset of drug administration	From cessation of drug administration	From onset of drug administration	From cessation of drug administration
Initial treatment	5–90 days	<5 or >90 days	≦15 days	—	>15 days
Subsequent treatment	1–15 days	>15 days	≦15 days	—	>15 days

**(c):** 

	Suggestive from onset of drug administration (score +2)	Compatible (score +1)		Incompatible (score 0)	
		From onset of drug administration	From cessation of drug administration	From onset of drug administration	From cessation of drug administration
Initial treatment	5–90 days	<5 or >90 days	≦30 days	—	>30 days
Subsequent treatment	1–90 days	>90 days	≦30 days	—	>30 days

**Table 2:** Category of groups.

	Category	Histological findings
Group A	DILI is strongly suspected	At least one of the pathological features suggesting DILI^*∗*^ was observed and all pathologists suspected DILI

Group B	DILI is suspected	At least one of the pathological features suggesting DILI^*∗*^ was observed

Group C	DILI should be considered in the differential diagnosis	Pathological features suggesting DILI^*∗*^ were not observed

^*∗*^Pathological features suggesting DILI were shown in Box 1.

DILI: drug-induced liver injury.

**Table 3:** Correlation between histologic groups and DDW-J scale for a diagnosis of DILI.

	Histologic groups for a diagnosis of DILI		
	A	B	C
DDW-J scale for a diagnosis of DILI			
Probable	●●●●●●●●●●●●●●●●●●●●●●●●●●●●	●●●●●●●●●●●●●●●●●●●●	
Possible	●●●●●●●●●●⋄●●●⋄●●●○●	○●○○⋄○○○○○○○○○○	●●●●⋄●●⋄●●○●
Unlikely	●●●●●●●●●●●●	●⋄○●●⋄○●●○○●●○○●●○○●	⋄○○○⋄○○○○○○○○○○○○○○○○○○○

Final clinical diagnosis of individual case: DILI ●, not DILI ○, and AIH ⋄.

DILI: drug-induced liver injury and AIH: autoimmune hepatitis.

**Table 4:** The histological patterns and clinical types according to DDW-J scale.

	The histological patterns			Total (cases)
	Hepatocellular type	Cholestatic type	Mixed type	
Clinical types according to the DDW-J scale				
Hepatocellular type	30	2	2	34
Cholestatic type	3	1	0	4
Mixed type	1	0	2	3

Total (cases)	34	3	4	41

**Table 5:** Two patients histologically classified as the cholestatic type but clinically classified as the hepatocellular type.

Gender	Age	The histological examination	DDW-J scale scores	The histological patterns	Clinical types according to DDW-J scale	Medicines which have been taken	DLST	ALT (IU/L)	AST (IU/L)	ALP (IU/L)	T-bil (mg/dL)
M	62	A	4	Cholestatic type	Hepatocellular type	Prednisolone, tranilast, teprenone	Negative	591	189	1003	7.4

F	37	A	6	Cholestatic type	Hepatocellular type	Phenytoin, famotidine, valaciclovir, lorazepam, and etizolam	Negative	718	331	616	1.9

DLST: drug-lymphocyte stimulation test.

**Table 6:** Three patients histologically classified as the hepatocellular type but clinically classified as the cholestatic type.

Gender	Age	The histological examination	DDW-J scale scores	The histological patterns	Clinical types according to DDW-J scale	Medicines which have been taken	Underlying disease	DLST	ALT (IU/L)	AST (IU/L)	ALP (IU/L)	T-bil (mg/dL)
M	83	A	5	Hepatocellular type	Cholestatic type	Meloxicam, bucillamine	RA	Negative	161	231	1268	1.4

F	49	B	5	Hepatocellular type	Cholestatic type	Alendronate sodium hydrate, methotrexate, and sodium ferrous citrate	RA	Not available	97	102	766	0.6

F	55	B	3	Hepatocellular type	Cholestatic type	Candesartan, trichlormethiazide, miglitol, and rabeprazole sodium	DM HT	Negative	168	152	1350	1.0

RA: rheumatoid arthritis, DM: Diabetes Mellitus, and HT: hypertension.

## References

[1] Fontana R. J., Seeff L. B., Andrade R. J. (2010). Standardization of nomenclature and causality assessment in drug-induced liver injury: summary of a clinical research workshop. *Hepatology*.

[2] Ostapowicz G., Fontana R. J., Schioødt F. V. (2002). Results of a prospective study of acute liver failure at 17 tertiary care centers in the United States. *Annals of Internal Medicine*.

[3] Chalasani N., Björnsson E. (2010). Risk factor for idiosyncratic drug-induced liver injury. *Gastroenterology*.

[4] Lucena M. I., García-Cortés M., Cueto R., Lopez-Duran J., Andrade R. J. (2008). Assessment of drug-induced liver injury in clinical practice. *Fundamental & Clinical Pharmacology*.

[5] Lewis J. H., Kleiner E., Burt A. D., Portmann B. C., Ferrel L. D. (2006). Hepatic injury due to drugs, chemicals and toxin. *MacSween's Pathology of the Liver*.

[6] Tajiri K., Shimizu Y. (2008). Practical guidelines for diagnosis and early management of drug-induced liver injury. *World Journal of Gastroenterology*.

[7] Watanabe M., Shibuya A., Miura Y. (2007). Validity study of DDW-J2004 scoreing scale for drug-induced liver injury. *Kanzo*.

[8] Hanatani T., Sai K., Tohkin M. (2014). A detection algorithm for drug-induced liver injury in medical information databases using the japanese diagnostic scale and its comparison with the council for international organizations of medical sciences/the roussel uclaf causality assessment method scale. *Pharmacoepidemiology and Drug Safety*.

[9] Takikawa H., Onji M., Takamori Y. (2005). Proposal of diagnostic criteria of drug induced hepatic injury in DDW-J2004 workshop. *Kanzo*.

[10] Nakanuma Y., Mukai K., Manabe T., Fukayama M. (2006). Liver. *Surgical Pathology*.

[11] Fukusato T., Oobe M., Nakanuma Y. (2013). Drug-induced liver injury. *Basic and Practical Liver Pathology*.

[12] Nakanuma Y., Harada K., Ren X. S. (2009). Drug-induced liver injury, pathology. *Pathology and Clinical Medicine*.

[13] Scheuer J. P., Lefkowitch J. H. (2010). Drugs and toxins. *Liver Biopsy Interpretation*.

[14] Fontana R. J., Watkins P. B., Bonkovsky H. L. (2009). Drug-Induced Liver Injury Network (DILIN) prospective study: rationale, design and conduct. *Drug Safety*.

[15] Kleiner D. E., Chalasani N. P., Lee W. M. (2014). Hepatic histological findings in suspected drug-induced liver injury: systematic evaluation and clinical associations. *Hepatology*.

[16] Björnsson E., Talwalkar J., Treeprasertsuk S. (2010). Drug-induced autoimmune hepatitis: clinical characteristics and prognosis. *Hepatology*.

[17] Suzuki A., Brunt E. M., Kleiner D. E. (2011). The use of liver biopsy evaluation in discrimination of idiopathic autoimmune hepatitis versus drug-induced liver injury. *Hepatology*.

[18] Davern T. J., Chalasani N., Fontana R. J. (2011). Acute hepatitis E infection accounts for some cases of suspected drug-induced liver injury. *Gastroenterology*.

